# The Current Landscape of Robotics in Lymphatic Supermicrosurgery: A Systematic Review

**DOI:** 10.1055/a-2844-9803

**Published:** 2026-05-29

**Authors:** Jorge Flores Garcia, Anne Huang, Joon Pio Hong, Min-Jeong Cho

**Affiliations:** 1 Department of Plastic and Reconstructive Surgery, The Ohio State University Wexner Medical Center, Columbus, Ohio, United States; 2 Department of Plastic and Reconstructive Surgery, Asan Medical Center, Seoul, Korea

**Keywords:** robotic-assisted lymphatic surgery, robotic-assisted microsurgery, lymphatic surgery, Symani, MUSA

## Abstract

**Background:**

Lymphedema affects over 250 million people worldwide and is increasingly managed with physiologic procedures such as lymphovenous bypass (LVB) and vascularized lymph node transfer (VLNT). These supermicrosurgical techniques require advanced training, are ergonomically demanding, and can be limited in anatomically constrained regions. Robotic surgical systems offer high-resolution visualization, motion scaling, tremor reduction, and improved ergonomics, making them promising adjuncts for lymphatic supermicrosurgery. However, evidence on safety, feasibility, and outcomes remains limited.

**Methods:**

A systematic review was conducted in accordance with PRISMA guidelines using PubMed, Cochrane Library, and Embase from inception through December 2024. Eligible studies included human clinical reports of robotic-assisted peripheral lymphatic reconstruction. Extracted data included demographics, operative details, outcomes, and study quality.

**Results:**

Seven studies involving 134 patients and 221 anastomoses were included, of which 78% (172) were performed robotically. The Symani system was most frequently used. Robotic procedures comprised 59.9% LVBs and 32% VLNTs or other free flaps. Across studies, mean anastomotic time was longer with robotic assistance (25.7 minutes) compared with manual techniques (11 minutes); however, three studies demonstrated significant time reduction with experience, ultimately approaching manual times. When reported, clinical outcomes, including limb volume reduction and quality-of-life measures, were comparable between robotic and manual groups. Advantages included tremor elimination and improved ergonomics, while challenges included cost, setup time, and lack of haptic feedback.

**Conclusions:**

Robotic-assisted lymphatic supermicrosurgery is safe and feasible, with outcomes comparable to conventional surgery. Continued refinement, cost reduction, and standardized outcome reporting will determine its role in expanding therapeutic and preventive lymphatic reconstruction.

## Introduction

Lymphedema is a chronic, progressive, and debilitating disease that affects an estimated 250 million people worldwide. 1 While currently there is no cure, physiologic procedures including lymphovenous bypass (LVB) and vascularized lymph node transfer (VLNT) are effective interventions that address the underlying diseased lymphatic system. 2 However, these procedures are technically challenging and require specialized expertise in supermicrosurgery, performing microanastomosis in vessels smaller than 0.8 mm. The increasing demand for preventative and physiologic lymphatic surgery highlights the growing need for ergonomic, high-precision surgical solutions, particularly in deep or anatomically constrained fields. 3 Robotic surgical systems are well positioned to meet this demand by enhancing the magnification and resolution of the surgical field, reducing tremor and human error, improving surgeon ergonomics, and supplementing surgical education. 4 5 6

Despite the popularity of robotic surgical systems in other surgical specialties for decades, they were not applied to microsurgery until 2006 with the first arterial anastomosis in a human performed using the Da Vinci robot (Intuitive Surgical Inc., Sunnyvale, USA). 7 Although early cases were successful, it quickly became clear that the Da Vinci system lacked the specifications required for microsurgery such as microsurgical instruments and adequate image magnification. 8 To address these concerns, two microsurgical robotic systems have been developed and approved for clinical use: the MUSA (MicroSure, Eindhoven, The Netherlands) and the Symani Surgical System (Medical Microinstruments, Inc., Wilmington, USA). These robotic systems have been adopted in a variety of microsurgical cases, including head and neck, brachial plexus, and autologous breast reconstruction. 9 However, there are limited studies on its use in lymphatic surgery. 4 10 This systematic review investigates the feasibility and clinical outcomes of robotic-assisted lymphatic surgery (RALS) for peripheral lymphedema treatment. In addition, we share future perspectives on the emerging role of robotic-assisted microsurgery.

## Materials and Methods

**Fig. 1:**
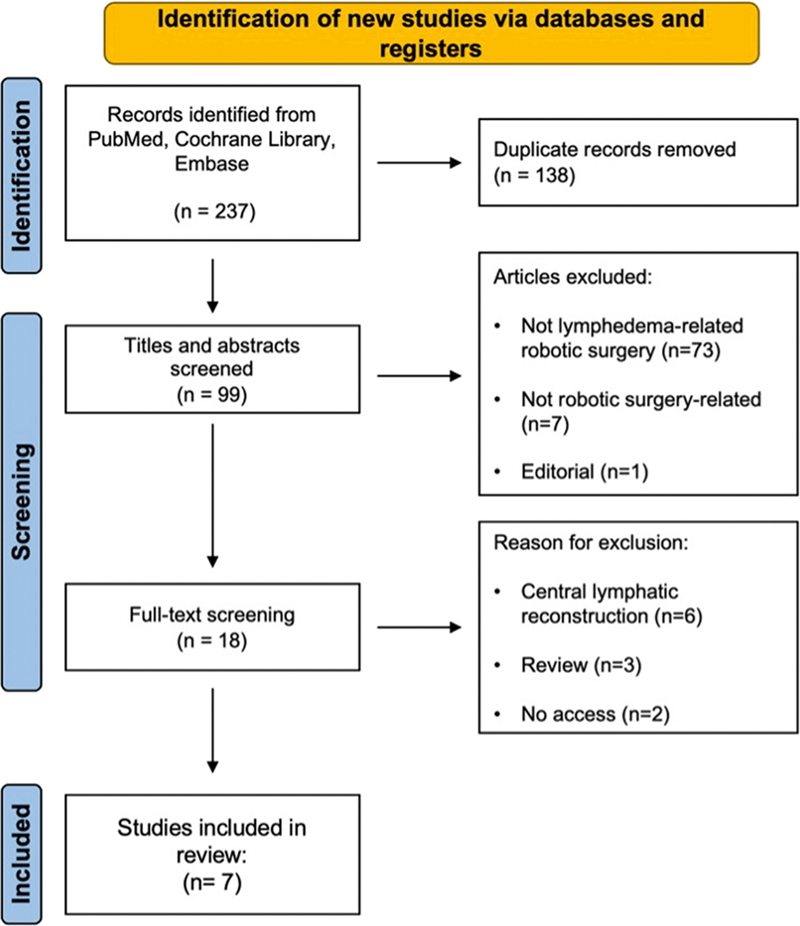
Preferred reporting items for systematic reviews and meta-analyses (PRISMA) flow diagram.

A review of the literature following the Preferred Reporting Items for Systematic Reviews and Meta-Analyses (PRISMA) guidelines for systematic reviews was conducted to investigate robotic-assisted microsurgery in peripheral lymphatic reconstruction ( Fig. 1 ). The search was conducted using PubMed, Cochrane Library, and Embase databases and encompassed all studies published from database inception to December 2024. The following terms were used: “robot” or “robotic assisted microsurgery” combined with “lymphatic reconstruction,” “lymphovenous bypass,” “lymphovenous anastomosis,” “lymph node transfer,” “LYMPHA,” “Symani AND lymphatic,” or “Microsure AND lymphatic.”

All studies published prior to the date of the search were screened for eligibility based on their title and abstract content. Screening and data extraction were performed independently by two reviewers (J.F.G. and A.H.). Studies on robotic-assisted peripheral lymphatic surgery were included. Exclusion criteria included: (1) non-English, (2) non-human, (3) studies not related to peripheral lymphatic reconstruction, and (4) review articles. Additionally, conference abstracts, unpublished or non–peer-reviewed material, and editorials were excluded. Data regarding study type, patient characteristics (age, gender, BMI, lymphedema type, International Society of Lymphology [ISL] stage, and site), operative details (robotic system, physiologic procedure, and operative times), and clinical outcomes (objective and patient reported) were extracted when provided. Randomized control trials were evaluated using the Cochrane Risk of Bias 2 (RoB-2) tool to assess bias in randomization, deviations from intended interventions, missing data, measurement of outcomes, and selective reporting. 11 Non-randomized and observational studies were assessed using the Methodological Index for Non-Randomized Studies (MINORS) and were scored based on eight criteria for non-comparative studies (maximum score: 16) and 12 criteria for comparative studies (maximum score: 24). 12 For each included study, reported funding sources and author conflict-of-interest (COI) disclosures were reviewed. Studies were not excluded solely on the basis of industry funding or disclosed relationships; however, such disclosures were considered during risk-of-bias assessment. The RoB-2 tool and the MINORS criteria were applied to evaluate methodological rigor independent of funding status.

## Results

### Search Results and Cohort Characteristics

**Fig. 2:**
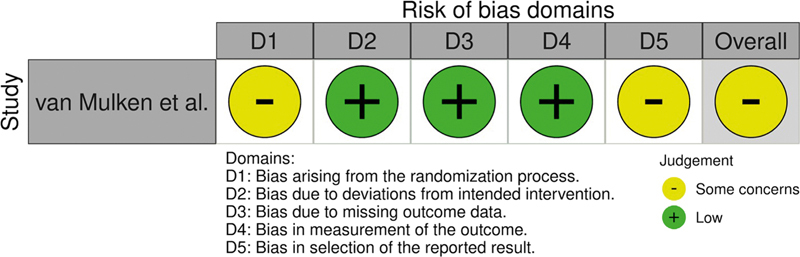
Cochrane risk of bias tool for randomized trials.

The initial search yielded 237 articles. Following removal of duplicates and review of titles and abstracts, 18 articles of potential relevance remained. Of these, 12 were excluded: primarily non-peripheral lymphatic reconstruction (6), reviews (3), and lack of access (2). Ultimately, seven studies were included: four with a retrospective design 13 14 15 16 and three with a prospective design. 17 18 19 Two studies were evaluated using the RoB-2 tool ( Fig. 2 ) while the remaining were assessed using the MINORS ( Table 1 ). COI disclosures were present in several studies. Specifically, author consultancy, advisory roles, or shareholder status in robotic microsurgical systems (MicroSure and Medical Microinstruments) were reported in six of the seven included studies. 13 14 15 17 18 19 Among the three prospective studies, one was a randomized controlled trial, 19 and the other was a 1-year follow-up of the same patient cohort. 18 Both studies from this cohort were included to ensure a comprehensive assessment of surgical and outcome measures. To account for patient overlap and data duplication, we considered these studies as one during data analysis. Perioperative variables and short-term outcomes were extracted from the original randomized pilot trial, whereas longer-term clinical outcomes were extracted exclusively from the 1-year follow-up publication. Four studies were exclusively on lymphedema patients, while the rest included patients undergoing other microsurgical procedures.

**Table 1:** Methodological index for non-randomized studies (MINORS)

Study	Study design	MINORS score
von Reibnitz et al, 2024 13	Retrospective	10
Reilly et al, 2024 17	Prospective	10
Weinzierl et al, 2023 14	Retrospective	10
Barbon et al, 2022 15	Retrospective	10
Lindenblatt et al, 2022 16	Retrospective	10

In total, there were 134 patients (81% female) ( Table 2 ). Five studies reported age, and two studies reported BMI, having a weighted mean age of 50.6 years and BMI of 25.3 kg/m ^2^ . About 82% of patients underwent surgery for lymphedema treatment, with etiology reported as primary lymphedema (21.6%), secondary lymphedema due to breast cancer treatment (35.1%), and undisclosed (43.3%). In five studies reporting lymphedema site, the upper extremities were most common (50%), followed by lower extremities (48%) and genital region (2%).

**Table 2:** Study cohort characteristics

Study	Study design	Robotic system used	Patient no.	Mean age, years	Mean BMI, kg/m ^2^	Lymphedema patients, *N* (%)	Lymphedema etiology, ( *N* [%])	Affected extremity, ( *N* [%])
von Reibnitz et al, 2024 13	Retrospective	Symani	67	48.3 (range 10–88)	—	55 (82.1%)	Primary (22 [40%]); secondary (33 [60%])	Upper (9 [16.4%]); lower (44 [80%]); genital (2 [3.6%])
Reilly et al, 2024 17	Prospective	MUSA-2	12	54.1 (SD 9.5)	27.8 (3.5)	12 (100%)	BCRL (12 [100%])	Upper (12 [100%])
Weinzierl et al, 2023 14	Retrospective	Symani	8	51.5 (range 20–62)	28.7 (range 19.7–42.7)	8 (100%)	Primary (1 [12.5%]); secondary—BCRL (7 [87.5%])	Upper (100%)
Barbon et al, 2022 15	Retrospective	Symani	22	46.1 (SD 19.2)	—	18 (81.8%)	Secondary—undisclosed (18 [100%])	—
Lindenblatt et al, 2022 16	Retrospective	Symani	5	50.8 (range 34–61)	—	3 (60%)	Primary (1 [33.3%]), secondary—undisclosed (2 [66.7%])	Lower (5 [100]%)
van Mulken et al, 2020, 2022 18 19	Randomized pilot trial, 1-year follow-up	MUSA	20	Robot: 60 (SD 11); manual: 60 (SD 7)	Robot: 27 (SD 11); manual: 25 (SD 5)	20 (100%)	Secondary—BCRL (20 [100%])	Upper (20 [100%])

Abbreviations: BCRL, breast cancer-related lymphedema; SD, standard deviation.

Note:— = not specified.

### Surgical Details

A total of 221 anastomoses were performed, with 78% robotically assisted (172 anastomoses). The Symani Surgical System was used in four studies, the MUSA in one, and the MUSA-2 in another. Robotic-assisted anastomoses included LVB (59.9%), arterial anastomoses (32%) for free flaps or VLNT, lympholymphatic anastomoses (2.3%), and epineural coaptation (1.2%) ( Table 3 ).

**Table 3:** Study surgical characteristics

Study	Anastomosis no.	Robotic anastomoses, anastomosis no. (%)	Robotic anastomosis type, (robotic anastomosis no. [%])	Manual anastomoses, anastomosis no. (%)	Manual anastomosis type, (manual anastomosis no. [%])	Additional lymphatic procedures, (procedure no.)	Mean total operative time, min	Mean robotic anastomosis time, min	Mean manual anastomosis time, min
von Reibnitz et al, 2024 13	100	100 (100%)	LVB (53 [53%]), arterial (44 [44%]), LLA (3 [3%])	—	—	Omental VLNT (46)	Robot: 365.3 (range 107–604)	—	—
Reilly et al, 2024 17	20	16 (80%)	LVB (16 [100%])	4 (20%)	LVB (4 [20%])	—	Robot: 212 (range 144–292)	32 for 1st anastomosis and 22.5 for 2nd in cases with 2 robotic anastomoses	15.5
Weinzierl et al, 2023 14	Not specified	All	LVB, arterial	—	—	Omental VLNT (9)	—	22.6 (range 10–59)	
Barbon et al, 2022 15	43	32 (74.4%)	LVB (20 [62.5%]); arterial (9 [28.1%]); epineural coaptation (2 [6.3%]); LLA (1 [3.1%])	11 (25.6%)	LVB (8 [72.7%]), arterial (3 [27.3%])	VLNT (UK)	—	25.3 (SD 12.3)	14.1 (SD 4.3) ^a^
Lindenblatt et al, 2022 16	18	10 (55.6%)	Lymphatic (8 [75%]); arterial (2 [25%])	8 (44.4%)	Lymphatic (7 [87.5%]); not specified (1 [12.5%])	Axillary VLNT (2)	Robot/manual: 349.6	—	—
van Mulken et al, 2020, 2022 18 19	40	14 (35%)	LVB (14 [100%])	26 (65%)	LVB (26 [100%])	—	Robot: 115 (range 69–115), manual: 81 (range 48–140)	25 (range 16–33) ^a^	9 (range 4–36) ^a^

Abbreviations: GA, general anesthesia; LLA, lympholymphatic anastomosis; LVB, lymphovenous bypass; UK, unknown; VLNT, vascularized lymph node transfer.

Note: ^a^ Significant difference in times.

Across four studies reporting robotic assistance for anastomoses exclusively, the average operative duration was 339.3 minutes (range 107–604 minutes) 13 16 17 18 while the average robotic anastomosis time in three studies was 25.7 minutes (range 10–59 minutes). In contrast, van Mulken et al reported a mean operative duration of 81 minutes for manual procedures, and a mean of 2.1 anastomoses per patient. 18 The average manual anastomosis time across three studies was 11 minutes (range, 8–36 minutes), with two studies demonstrating significantly shorter manual compared with robotic-assisted anastomosis times (manual: 14.1 and 9 minutes vs. robotic: 23.9 and 25 minutes). 15 19 Reilly et al reported the mean lumen diameter of lymphatic (0.49 mm) and venous (0.38 mm) vessels used for robotic anastomosis, 17 while two others reported diameters of 0.3 to 0.5 mm 14 and 0.8 mm 16 in individual cases.

Three studies demonstrated a learning curve with significant reductions in robotic anastomosis time as experience increased. Weinzierl et al reported a decrease from 59 minutes per anastomosis in the first patient to a mean of 20 minutes in the subsequent seven patients. 14 Van Mulken et al observed a reduction from 31 to 17 minutes between the first and fifth anastomoses. 19 Similarly, Barbon et al reported a decrease in mean robotic anastomosis time from 23.9 to 16.3 minutes between initial and late patient groups, ultimately achieving comparable manual and robotic times, with the final patient undergoing robotic LVBs in 10 to 12 minutes and a manual in 10 minutes. 15 Reilly et al additionally reported reductions in surgeon-reported effort and frustration related to robotic surgery over time despite no meaningful reduction in anastomotic time. 17 Furthermore, operative and set-up times decreased as the surgical team became familiar with the robotic system.

### Outcomes

There was substantial heterogeneity in study design and reporting of outcomes ( Table 4 ). Only three studies reported limb volume outcomes, two of which also assessed quality of life measures ( Supplemental Table S1 ). 13 14 18 Von Reibnitz et al reported a volume reduction of 281 mL (7.6%) in 86% of upper extremity patients ( *N* = 7) and 288 mL (1.4%) in 72% of lower extremity patients ( *N* = 32) at 3 months. 13 In another study, Weinzierl et al described a single BCRL patient who experienced a 25.2% (936 mL) volume decrease in the affected arm compared with the unaffected and 100% excess limb volume reduction following a VLNT. 14

**Table 4:** Study outcomes

Study	Mean follow-up	Volume outcomes	Patient-reported outcomes	Additional outcomes	Complications	Robot benefits	Robot challenges
von Reibnitz et al, 2024 13	10.1 months (range 0–26)	Mean 7.6% volume reduction in 6/7 UE, mean 1.4% volume decrease in 23/32 LE	—	—	6 patients with wound infections, 2 complicated by lymphocele and wound dehiscence, 1 hematoma, and 1 case of wound dehiscence without revision	Motion scaling reduces tremor, improved precision, ergonomics	Increased operative times, higher costs, difficulties with setup, additional training required for surgical and technical staff
Reilly et al, 2024 17	—	—	—	—	—	—	Suture broke in 75% of cases, high surgeon-reported frustration and effort early on
Weinzierl et al, 2023 14	—	—	—	—	—	Setup used improved surgeon's positioning and allowed independent functioning without assistant	—
Barbon et al, 2022 15	—	—	—	—	Seroma formation after 4 sarcoma resections (not related to the robot); thrombosis of an irradiated vessel for which Symani was used	Performing LVB of vessels with significant size mismatch, facilitates preventive LVB in deep proximal thigh, intuitive nature of robot	Longer operative times, higher costs, instrument stickiness
Lindenblatt et al, 2022 16	—	—	—	—	—	High accuracy in placing stitches in fragile vessels within small spaces, performing anastomoses of vessels with size mismatches	Lack of haptic feedback, necessity to develop a “see feel” with eyes, setup issues and instrument stickiness in early cases
van Mulken et al, 2020, 2022 18 19	Robot: 378 days; manual: 376 days	Non-significant UELI increase in robot (+2.77) and manual (+3.31) in unaffected vs. affected arm and in general UELI (+6.99 & +7.2)	Decrease in Lymphedema, Disability, and Health Questionnaire scores in robot (20 points) ^a^ and manual (23 points) groups ^a^	66% robot vs. 81.8% manual had patent anastomosis at 1 year; ADB improvement in 6 patients (5 manual, 1 robot), worsening in 1 robotic anastomosis	4 cases of erysipelas in 3 patients	—	When graded by 2 blinded microsurgeons, manual anastomosis quality was greater vs. robotic

Abbreviations: ADB, arm dermal backflow stage; LE, lower extremity; LLA, lympholymphatic anastomosis; LVB, lymphovenous bypass; UELI, upper extremity lymphedema index; VLNT, vascularized lymph node transfer.

Note: ^a^ Significant finding.

The randomized pilot trial and 1-year follow-up studies cohort provided the most robust outcomes, with results including upper extremity lymphedema index (UELI) as a measure of volume, 20 anastomotic patency rates, arm dermal backflow (ADB) staging, and patient reported outcomes using the Lymphedema Functioning, Disability, and Health Questionnaire (Lymph-ICF) 1 year postoperatively. 18 19 A greater proportion of patients in the manual group demonstrated a single patent anastomosis (81.8% vs. 66.6%) and improvement in ADB staging compared with the robotic-assisted group. Despite these findings, no intervention demonstrated superiority in terms of mean UELI at 1, 3, 6, and 12 months. Two cases of erysipelas occurred in the robotic-assisted group, one of which occurred in a patient with a prior history of recurring erysipelas.

Furthermore, this study observed significant quality of life improvements in both groups, with mean Lymph-ICF score reductions of 20 points ( *p* = 0.045) in robotic-assisted patients and 23 points ( *p* = 0.001) in manual patients. Decreased compression use was noted in 42.9% of robotic-assisted and 45.5% of manual patients, while frequency of manual lymphatic drainage decreased in the manual group (6.5 to 2.92 per month) and increased in the robotic-assisted (3.69 to 3.75). Similar to UEL, no significant intervention effect was found on quality of life at any time point.

## Discussion

### Clinical Outcomes of Robotic-assisted Lymphedema Surgery

This systematic review of seven studies on RALS demonstrates its safety and feasibility in lymphatic reconstruction. Across the included studies, a total of 172 robotic-assisted microsurgical anastomoses (78% of 221 anastomoses reported) were performed. Of these, 64.5% were LVBs and 32% were VLNTs or undisclosed free flaps. Among studies reporting operative details, the average robotic-assisted anastomosis time was 25.7 minutes (range, 10–59 minutes), compared with an average manual anastomosis time of 11 minutes (range, 8–36 minutes). Although longer anastomotic times have been cited as a limitation of robotic microsurgery, three studies demonstrated significant reductions in robotic anastomosis time with experience, and one study reported comparable manual and robotic anastomosis times over an 8-month study period. Consistent with these findings, a systematic review of robotic microsurgery across arterial, venous, nerve, and lymphatic anastomoses reported a mean improvement of 41.8% in anastomosis time with experience. 21 It is important to note that total operative duration varied substantially across studies, in part because some investigations included additional procedures such as VLNT or flap-based reconstruction in addition to lymphatic surgery; therefore, aggregate operative time estimates should be interpreted descriptively rather than as direct comparisons of procedural efficiency. Similarly, reported mean anastomotic times should be interpreted cautiously, as several studies did not stratify anastomosis duration by procedure type (i.e., supermicrosurgical LVB versus arterial or flap-related microanastomoses).

In terms of clinical outcomes, the randomized pilot trial by van Mulken et al demonstrated improvements in upper extremity lymphedema index and patient-reported outcomes in both robotic-assisted and manual groups; however, no intervention emerged as superior at any postoperative time point. 18 It is worth emphasizing that the clinical outcomes found in this study were limited, not standardized, and had short follow-up times. Additionally, these outcomes may be influenced by the fact that the studies report early institutional and surgeon experience with RALS. It is possible that increased experience and familiarity with robotic systems may lead to technical refinements, shorter operative times, improved ability to perform LVBs in technically challenging sites, and improved patient outcomes. Future studies should prioritize standardized objective and subjective outcomes with longer-term follow-up, as well as operative metrics such as anastomosis time, number and location of anastomoses, and surgeon ergonomics. Altogether, the available evidence supports the technical feasibility and short-term safety of RALS but does not permit conclusions regarding clinical efficacy, equivalence, or superiority.

### Expanding Indications of Robotic-assisted Lymphedema Surgery

One of the primary advantages of RALS is improved accessibility in anatomically constrained operative fields. Limited exposure and restricted instrument maneuverability can make supermicrosurgical lymphatic reconstruction technically challenging, particularly in deeper regions of the extremities, axilla, and head and neck. By enhancing precision and access in such environments, robotic-assisted systems may broaden the anatomical regions in which lymphatic supermicrosurgery can be performed.

Beyond peripheral lymphatic reconstruction, early reports suggest potential technical feasibility of robotic-assisted approaches in central lymphatic reconstruction. In 2023, Grünherz et al reported the first successful clinical report of robotic-assisted central lymphatic reconstruction, by performing anastomosis of a dilated, retroperitoneal lymphatic cyst to the left ovarian vein. 22 The same group also published their series of robotic-assisted microanastomosis of the thoracic duct to a nearby vein in the neck, thorax, or abdomen in 11 patients with acquired or congenital thoracic duct lesions, demonstrating feasibility and clinical improvement in 9 out of 11 patients. 23 The authors highlight improved access and precision in deep body cavities as key advantages, with particular relevance in congenital cases where smaller vessel size further increases technical difficulty. While additional studies from other institutions are necessary, these early reports suggest potential feasibility of robotic-assisted approaches for this rare and undertreated disease process.

More recently, the indications for physiologic lymphedema surgery have expanded to include prevention, such as immediate lymphatic reconstruction (ILR) or prophylactic LVBs performed at the time of lymph node dissection. 24 One of the technically challenging aspects of ILR is optimizing the surgical setup. ILR is frequently performed in anatomically deep or constrained spaces, such as the axilla, where supermicrosurgical precision can be technically demanding. To date, there are no studies evaluating robotic-assisted systems for ILR, and this application remains an area for future investigation. As the clinical significance of preventative lymphedema surgery continues to grow, further studies will be required to determine whether robotic microsurgical systems can meaningfully address the technical and ergonomic challenges associated with these procedures.

### Role of Robotic-assisted Lymphedema Surgery in Surgical Education

Robotic microsurgical systems may help expedite microsurgical training and increase the number of surgeons and medical centers able to perform supermicrosurgery, thereby increasing patients' access to lymphatic reconstruction. 25 Lymphatic supermicrosurgery is technically demanding, requiring specialized instruments and ultrafine 11–0 sutures with a diameter of 0.01 mm. It is also generally accepted that the outcomes of LVB are tied to the quality of supermicrosurgical anastomosis, requiring precision and accuracy at the limits of human ability. Robotic microsurgical systems may help mitigate these challenges through motion scaling and tremor elimination, as well as improved visualization with wider fields of view and greater depth of focus, reducing the need for frequent repositioning. Moreover, specialized instruments, such as self-cutting needle holders and combined instruments with more degrees of articulation, may reduce the need for a surgical assistant, allowing trainees to focus on performing the procedure rather than assisting. 4

While the learning curve of robotic microsurgery is steep, preclinical studies suggest that novice microsurgeons may achieve technical competency in robotic-assisted anastomoses more rapidly than with conventional hand-sewn techniques. 26 Prior microsurgical experience has been found to be helpful but not necessary to quickly achieve proficiency. 27 However, these early findings are derived primarily from bench-top and preclinical training models, and objective educational outcome metrics, such as error rates, precision, or time-to-proficiency, remain to be evaluated systematically in clinical training settings. Moreover, current studies of robotic-assisted surgical education have not specifically examined its application to lymphatic supermicrosurgery, but it would intuitively seem that the benefits of robotic systems are magnified on sub-millimeter vessels. As such, the educational role of robotic microsurgical systems remains largely theoretical, and further investigation using validated clinical and simulation-based models is needed to define their impact on training outcomes.

### Current Limitations

Current limitations to widespread adoption of RALS include substantial upfront capital investment, ongoing service and consumable costs, additional setup time during early implementation, training requirements for surgeons and operating room staff, and the absence of native haptic feedback. The financial impact is highly volume-dependent: prior cost analyses of robotic surgical programs demonstrate that fixed implementation and maintenance costs account for a large proportion of robotic-specific expenditures but may be amortized with high-volume utilization across multiple specialties, whereas per-case consumables remain a recurring expense. 28 29 For dedicated microsurgical platforms, reported system costs range from approximately $1 to 1.5 million, with additional annual maintenance costs exceeding $100,000 and instrument replacement costs of $2,000 to 10,000 per case, reinforcing that institutional adoption may depend on shared access across microsurgical services rather than lymphedema volume alone. 30 To date, no published studies have directly compared the costs of manual versus RALS or the financial impact on patients.

### Conclusions

This systematic review demonstrates the safety, feasibility, and early but heterogeneous outcomes reported in robotic-assisted lymphatic reconstruction for peripheral lymphedema. Future studies should investigate standardized clinical outcomes with longer follow-up. Other research interests include its impact on operative details, surgeon ergonomics, surgical education, and expanding indications.
