# Supplementary material for: The Current Landscape of Robotics in Lymphatic Supermicrosurgery: A Systematic Review
Source: Arch Plast Surg. 2026 May 29;53(3):267–74. doi: 10.1055/a-2844-9803 (PMC13287940; doi:10.1055/a-2844-9803)
Supplement: Supplementary file 1 — Supplementary Material [file 10-1055-a-2844-9803-s25sep0141oa.pdf]

**Supplemental Table 1.** Study reported postoperative outcomes measures

| Study | Mean Followup | Postoperative Outcome Measure |
|---|---|---|
| von Reibnitz *et al.* 2024 [13] | 10.1 months (range 0-26) | Limb volume measurement, compression garment use, complications |
| Reilly *et al.* 2024 [17] | --- | Patient satisfaction scores |
| Weinzierl *et al.* 2023 [14] | --- | --- |
| Barbon *et al.* 2022 [15] | --- | Complications |
| Lindenblatt *et al.* 2022 [16] | --- | --- |
| van Mulken *et al.* 2020, 2022 [18,19] | Robot: 378 days; Manual: 376 days | UELI, ADB stage, anastomosis patency (ICG), Lymphedema Functioning, Disability, and Health Questionnaire, compression garment use, MLD frequency, complications |

--- =not reported; UELI=upper extremity lymphedema index; ADB=arm dermal backflow; ICG=indocyanine green; MLD=manual lymphatic drainage
